# Benthic Composition of a Healthy Subtropical Reef: Baseline Species-Level Cover, with an Emphasis on Algae, in the Northwestern Hawaiian Islands

**DOI:** 10.1371/journal.pone.0009733

**Published:** 2010-03-17

**Authors:** Peter S. Vroom, Cristi L. Braun

**Affiliations:** Coral Reef Ecosystem Division, Joint Institute for Marine and Atmospheric Research, National Oceanic and Atmospheric Administration (NOAA) Fisheries, Pacific Islands Fisheries Science Center, Honolulu, Hawai'i, United States of America; University of Canterbury, New Zealand

## Abstract

The Northwestern Hawaiian Islands (NWHI) are considered to be among the most pristine coral reef ecosystems remaining on the planet. These reefs naturally contain a high percent cover of algal functional groups with relatively low coral abundance and exhibit thriving fish communities dominated by top predators. Despite their highly protected status, these reefs are at risk from both direct and indirect anthropogenic sources. This study provides the first comprehensive data on percent coverage of algae, coral, and non-coral invertebrates at the species level, and investigates spatial diversity patterns across the archipelago to document benthic communities before further environmental changes occur in response to global warming and ocean acidification. Monitoring studies show that non-calcified macroalgae cover a greater percentage of substrate than corals on many high latitude reef sites. Forereef habitats in atoll systems often contain high abundances of the green macroalga *Microdictyon setchellianum* and the brown macroalga *Lobophora variegata*, yet these organisms were uncommon in forereefs of non-atoll systems. Species of the brown macroalgal genera *Padina*, *Sargassum*, and *Stypopodium* and the red macroalgal genus *Laurencia* became increasingly common in the two northernmost atolls of the island chain but were uncommon components of more southerly islands. Conversely, the scleractinian coral *Porites lobata* was common on forereefs at southern islands but less common at northern islands. Currently accepted paradigms of what constitutes a “healthy” reef may not apply to the subtropical NWHI, and metrics used to gauge reef health (e.g., high coral cover) need to be reevaluated.

## Introduction

The Papahānaumokuākea Marine National Monument (PMNM) is among the largest marine protected areas in the world [1] and provides unprecedented opportunities to study intact subtropical reef ecosystems. Research in recent years has documented that reefs in the PMNM differ from many truly tropical, equatorially-situated reefs that typically contain island-wide averages of over 30% live coral cover [2]–[4] and instead are characterized by benthic communities containing patches of dense coral (up to 80% cover) interspersed amongst expansive stretches of hard-bottomed habitats dominated by algal functional groups [2], [5]. Despite these differences in coral cover, a key attribute of both healthy tropical and subtropical reef ecosystems is that they continue to contain thriving fish communities dominated by top predators [6]–[8].

Benthic communities in the PMNM represent a paradox: despite global recognition as being among the reefs least impacted by human activities on the planet [1], [9]–[11], direct anthropogenic stressors such as marine debris [12]–[14] and indirect human stressors such as coral bleaching [14]–[19] indicate that these reefs are at risk. Although still considered healthy, the current reef state may not reflect pre-human contact [20]. Future coring studies to examine historical reef assemblages [21] may aid in determining the historical diversity of calcifying organisms in the NWHI, but would not necessarily provide insight into the percent cover of living calcified organisms at any given time, and would not be a good indicator of the types of non-calcifying algal assemblages that might have been present in past eras. Also, normal oscillations in reef assemblages would have occurred due to changing sea levels and warming/cooling events over geologic time scales [22], and it cannot be assumed that one constant state of “reef health” was maintained over thousands of years [23]. Because of these issues, accurately defining pre-human reef attributes is difficult, but gaining current comprehensive, multidisciplinary, species-level surveys of benthic communities in the Northwestern Hawaiian Islands (NWHI) provides a critical baseline of the present state of NWHI reefs and will allow future researchers to determine if benthic communities change over time in the face of rapidly changing sea surface temperatures [24] and ocean acidification [25], [26].

To assess and monitor remote US Pacific reefs, the National Oceanic and Atmospheric Administration's (NOAA) Pacific Islands Fisheries Science Center's (PIFSC) Coral Reef Ecosystem Division (CRED) began quantitative, interdisciplinary, ecosystem-based monitoring in 2000 [27]. This research has led to detailed descriptions of coral community percent coverage and diversity for most islands located in the PMNM [28]–[34], but information about the non-coral organisms that dominate the majority of the substrate is less prevalent. Although in situ observations of algal assemblages in the NWHI occurred concurrently with coral surveys via a photoquadrat methodology from 2002 to 2007 [35], percent cover analyses of species level macroalgal populations have only been completed for three islands [5], [36], [37]. Nevertheless, in situ observations of the relative abundance of macroalgae (RAM) across the entire archipelago have served as a proxy for percent coverage data and have proven useful for documenting changes to algal assemblages in response to coral bleaching events [38].

Recent research [2], [3], [5], [37], [39], [40] has shown that current models linking large macroalgal populations with the deteriorated health of reefs were likely developed in response to massive coral to macroalgal phase shifts that occurred in the Caribbean [41], and oversimplify the essential role of macroalgae in healthy ecosystems [42]. In some unimpacted reef systems, macroalgal populations may occur in equilibrium with coral species and serve as critical habitat necessary for overall reef health. Thriving algal assemblages in reef ecosystems are often extremely diverse, and changes in macroalgal community dynamics (e.g., one macroalgal species being completely replaced by another) can occur without changes in coexisting coral assemblages [3]. Thus, it is imperative to examine percent coverage of all organisms, not just coral, at the species rather than functional group level to understand whether seasonal or permanent changes to reef systems are occurring.

In 2008, a line-point intercept (LPI) method [43] was instigated by CRED for determining species-level percent coverage of benthic organisms. This methodology provided data that were immediately available for analysis at the return of the research expedition. The ultimate goals of this study were threefold: (1) to make available vitally important, species-level baseline data from 62 long-term monitoring sites in the literature so that future researchers will have solid data for comparison if reef composition changes in response to global climate change or other anthropogenic factors, (2) to test the degree of benthic heterogeneity that exists at sites sampled across the NWHI by statistically comparing species-level percent cover among hierarchical scales (habitat type, geographic location in a reef system (quadrant), and islands) [44], [45] and, perhaps most importantly, (3) to demonstrate that healthy sub-tropical communities naturally contain a mix of both coral and algal dominated environments. Large expanses of macroalgal meadows are not indicative of decreased ecosystem health in the NWHI.

## Materials and Methods

### Background

**Figure 1:**
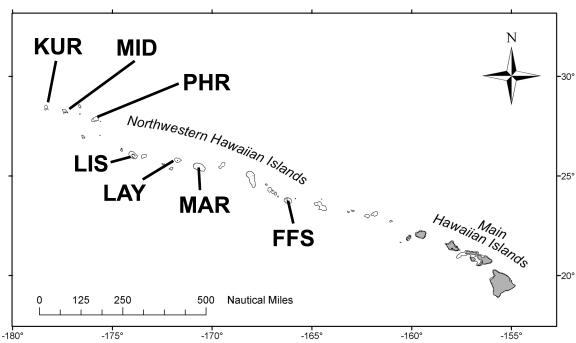
Map of the Northwestern Hawaiian Islands. KUR = Kure Atoll, MID = Midway Atoll, PHR = Pearl and Hermes Atoll, LIS = Lisianski Island, LAY = Laysan Island, MAR = Maro Reef, FFS = French Frigate Shoals.

The PMNM contains subtropical reef ecosystems scattered among 10 islands and atolls that stretch 2030-km to the northwest of the eight main Hawaiian Islands (Fig. 1). In 2003, an interdisciplinary team of scientists from CRED, the Northwestern Hawaiian Islands Coral Reef Ecosystem Reserve (a precursor to PMNM), the University of Hawai'i, and the Oceanic Institute selected 74 long-term monitoring sites to be visited annually in order to monitor ecosystems temporally [10]. To be statistically comparable, most sites monitored were located at a median depth of ∼13.7-m (45′) unless only shallower depths were encountered (Table S1) and represented 3 broad habitat types: forereef, backreef, and lagoon. In 2006, stainless steel pins were installed at 5-m intervals along two 25-m transect lines at each monitoring site to create permanent transects. In 2008, species-level percent cover observations were collected at 62 of these established monitoring sites representing 7 islands as part of the NWHI Reef Assessment and Monitoring Program (RAMP) research cruise between 14 September and 9 October (Table S1).

### Benthic sampling

Benthic communities were sampled along two 25-m transect lines set in a single file row (separated by 5-m) along permanent transect pins (Table S1), and organisms were recorded at 20-cm intervals using LPI methodologies (125 points/transect, 250 points/site) where macroscopic scleractinian corals, zooanthids, macroalgae, and macroinvertebrates were identified to the species level (when possible), and organisms difficult to taxonomically identify in the field (turf algae, cyanobacteria, and coralline red algae) were identified to the functional group level. Macroalgal species not identifiable in the field were collected, frozen, and identified in the Phycology laboratory at CRED using a Nikon E400 compound microscope (Nikon, Melville, NY, USA).

### Statistical analyses

The two 25-m transects surveyed at each site were oriented in a single file row separated by 5-m, and are pseudo-replicates (essentially one 55-m transect was surveyed). Therefore, data from each transect line at each site were pooled and analyzed at the site rather than transect level, with each of the 62 sites considered as an independent replicate. Data were converted from actual counts (250 points sampled/site) to percent cover equaling 100%, and placed in a Microsoft Excel data matrix. A Bray-Curtis resemblance matrix was subsequently created using PRIMER-E v.6 with a fourth-root transformation of the data to lessen the influence of prevalent species and increase the weight of rare species. Three-way mixed model PERMANOVA (maximum permutations = 9999) was used to test each data set, with island (French Frigate Shoals, Maro Reef, Laysan Island, Lisianski Island, Pearl and Hermes Atoll, Midway Atoll, Kure Atoll), habitat type (fore reef, back reef, lagoon), and quadrant (NW, SW, NE, SE) provided as factors. Islands were selected as a factor to determine if benthic communities differed latitudinally or by island type (atoll vs. non-atoll); quadrants were selected as a factor to determine if prevailing wave energy affected benthic community composition (giant NW swells are typical in winter months while less intense S swells are more common during summer months [46], [47]); habitat was selected as a factor to determine whether benthic communities differed among major reef classifications around each island. When significant main test effects were discerned, pairwise tests were conducted to determine which factors were responsible for significance. Non-metric multidimensional scaling (nMDS) ordinations were used to visually depict relationships among sites. SIMPER analyses were conducted to examine the contribution to dissimilarity of individual species or functional groups.

## Results

Forty-one species of macroalgae, 23 species of scleractinian coral, 1 species of zooanthid (*Palythoa caesia*), 2 species of urchin (*Echinostrephus aciculatus* and *Echinometra mathaei*), and 3 species of taxonomically unidentified sponges were observed along transect lines at 62 sites in the NWHI during 2008 surveys (Tables S2, S3, S4). Green algae were the most diverse macrophytes with 18 species recorded, 5 of these species occurring in the genus *Halimeda* (Table S2). Fourteen red and 9 brown algal species were recorded along transect lines, respectively (Table S2). *Halimeda velasquezii* and the brown alga *Lobophora variegata* were the most common algal species across the NWHI, occurring at 74% and 79% of surveyed sites, respectively (Table S2).

Seventy percent of coral species observed belonged to just 4 genera: *Montipora* (5 species), *Pocillopora* (4 species), *Porites* (4 species), and *Acropora* (3 species) (Table S3). The remaining 7 species belonged to 5 additional genera. *Porites lobata* was the most common coral species across the NWHI, occurring at 73% of surveyed sites (Table S3).

### Percent cover

**Figure 2:**
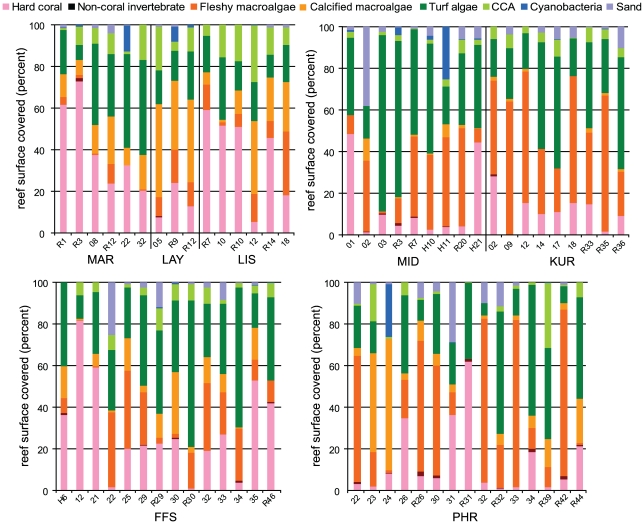
Percent cover of benthic functional groups at surveyed sites in the Papahānaumokuākea Marine National Monument. Median depth of ∼13.7-m. Each site consists of a single replicate, so error bars are not possible. Island abbreviations are the same as in Fig. 1. CCA = crustose coralline red algae.

Macroalgal assemblages (including both calcified and non-calcified species) covered more substrate than coral assemblages at 65% of sites surveyed, with non-calcified “fleshy” macroalgae occupying more substrate than corals at 53% of surveyed sites (Fig. 2, Table S4). When both fleshy and calcified macroalgae were considered as a whole, no pattern of increasing or decreasing cover was discernable among islands based on latitude or island type (atoll vs. island); however, when non-calcified macroalgae were considered alone, a latitudinal gradient was apparent with 100% of sites at Kure Atoll (∼28°25′ N), 67% of sites at Midway Atoll (∼28°12′ N), and 60% of sites at Pearl and Hermes Atoll (∼27°50′ N) containing more fleshy macroalgae than coral (Figs. 1, 2, Table S4). At French Frigate Shoals (∼23°50′ N), the most southerly situated atoll system in the NWHI, only 43% of sites contained more non-calcified macroalgae than coral. The majority of sites surveyed at non-atoll systems contained a greater percent cover of coral than fleshy algae. No pattern of coral or macroalgal domination was observed among habitat types, with 66%, 79%, and 50% of forereef, backreef, and lagoonal sites containing a greater percent cover of macroalgae (both non-calcified and calcified) than coral, respectively (Figs 1, 2, Tables S1, S4).

Green algae were the most dominant macroalgae in the NWHI with the non-calcified foliose macroalga *Microdictyon setchellianum* averaging 13.1% cover (SE 2.4) when data from all 62 sites were pooled. *Microdictyon setchellianum* commonly formed meadows, and covered >10% of the substrate at 41% of sites surveyed (and up to 76% of the substrate in certain forereef areas at Pearl and Hermes Atoll and French Frigate Shoals) (Table S2). Species of the calcified genus *Halimeda* occupied >10% of the substrate at 34% of sites surveyed (Table S2) and averaged 9.5% cover (SE 1.7) when data from all 62 sites were combined. Brown algae, especially *Stypopodium flabelliforme* and species of *Padina* and *Sargassum* were minor components of benthic communities south of Pearl and Hermes Atoll, but became increasingly abundant (and sometimes dominant) in reef systems at Midway and Kure Atolls (Table S2). A similar trend was observed for the red alga, *Laurencia galtsoffii*, which achieved a percent cover exceeding 35% at one site at Midway Atoll (Table S2). Other red algal species were a common, but minor, component of reef systems in the NWHI.

*Porites lobata* occupied a greater amount of substrate than other coral species across the NWHI and exhibited percent cover of >10% at 29% of sampled sites. Converse to the distribution of *Stypopodium*, *Padina*, *Sargassum*, and *Laurencia*, *P. lobata* commonly occurred in forereef regions of reefs located south of Midway Atoll, but was a minor component of reefs at both Midway and Kure (Table S3). The table coral *Acropora cytherea* was only recorded at French Frigate Shoals (up to 80% cover at select sites) and was absent from sites surveyed at more northerly situated islands (Table S3).

### Multivariate analyses

PERMANOVA found significant differences to occur among benthic community composition at surveyed sites based on island and habitat (*p*-value<0.05; Table 1). Additionally, the interactions of island+habitat and habitat+geographic area (quadrant) were significant. Further pairwise tests (data not shown) revealed that similar habitat types at different islands supported different types of benthic communities; however, benthic communities from similar habitats but different quadrants within a single island ecosystem did not differ substantially.

**Table 1:** Results of PERMANOVA main test.

Source	df	SS	MS	Pseudo-F	P(perm)	Unique perms
Is	5	17329	3465.9	3.8505	0.0001	9893
Ha	1	3247.3	3247.3	3.6076	0.0021	9949
Qu	2	2010.4	1005.2	1.1168	0.3586	9935
IsxHa	6	10202	1700.4	1.8891	0.0037	9901
IsxQu	10	12160	1216	1.3509	0.0663	9869
HaxQu	4	8608.7	2152.2	2.391	0.0018	9917
IsxHaxQu	3	3326.9	1109	1.232	0.2425	9914
Res	26	23403	900.11			
Total	61	1.0056E5				

Is = Island, Ha = Habitat type (forereef, backreef, lagoon), Qu = Quadrant (NW, SW, NE, SE).

When benthic cover from forereef sites across different islands was compared, most atoll systems (French Frigate Shoals (FFS), Pearl and Hermes Atoll (PHR), Midway Atoll (MID), Kure Atoll (KUR)) were found to be statistically similar (the exceptions being the FFS to KUR and PHR to MID comparisons) and to typically contain high populations of *Microdictyon setchellianum* (6.3%–34.9%), *Lobophora variegata* (5.0%–10.7%), and turf algae (25.5%–48.6%) (Table 2). However, the majority of benthic communities at sites surveyed from forereef habitats on non-atoll islands were significantly different from forereef sites on the 4 atolls. SIMPER revealed the green macroalga *Microdictyon setchellianum* and the brown macroalga *Lobophora variegata* were not common components of surveyed sites in the forereef environments of the non-atoll systems of Maro Reef (MAR), Laysan Island (LAY), and Lisianski Island (LIS), whereas crustose coralline red algae (6.6% to 11.9%) were more prevalent at these non-atoll systems than on atoll forereefs (Table 2).

**Table 2:** Average percent cover of the six most dominant items occurring at each island by habitat type.

	Forereef		Backreef		Lagoon	
**FFS**	*n* = 4, ave. sim. = 54.20		*X* = 1		*n* = 9, ave. sim. = 43.84	
	turf algae	43.8 (6.8)	turf algae	70.4	turf algae	28.9 (3.3)
	*Microdictyon setchellianum*	12.8 (5.1)	*Microdictyon setchellianum*	14.4	*Acropora cytherea*	15.6 (9.6)
	*Lobophora variegata*	10.7 (3.5)	CCA	8	*Porites lobata*	13.9 (4.9)
	*Porites lobata*	9.7 (3.9)	*Lobophora variegata*	1.2	*Lobophora variegata*	7.4 (2.6)
	*Halimeda velasquezii*	6.9 (2.3)	Sand	0.8	*Halimeda velasquezii*	7.3 (3.0)
	*Pocillopora meandrina*	5.9 (2.6)			CCA	5.6 (1.0)
**Maro**	*n* = 6, ave. sim. = 51.44					
	turf algae	31.3 (6.2)	no backreef present		no lagoon present	
	*Porites lobata*	11.7 (2.7)				
	*Porites compressa*	11.5 (3.2)				
	*Montipora capitata*	10.1 (3.4)				
	*Halimeda velasquezii*	7.0 (2.0)				
	CCA	6.6 (2.4)				
**Laysan**	*n* = 3, ave. sim. = 67.33					
	*Halimeda velasquezii*	38.1 (3.2)	no backreef present		no lagoon present	
	turf algae	17.9 (2.7)				
	CCA	11.9 (4.9)				
	*Porites lobata*	10.1 (5.9)				
	*Laurencia majuscula*	7.5 (2.5)				
	*Pocillopora meandrina*	3.5 (1.9)				
**Lisianski**	*n* = 6, ave. sim. = 51.52					
	turf algae	18.2 (2.7)	no backreef present		no lagoon present	
	*Porites evermanni*	17.1 (5.9)				
	CCA	11.0 (2.0)				
	*Porites lobata*	11.0 (4.3)				
	*Halimeda opuntia*	9.5 (3.6)				
	*Halimeda velasquezii*	6.6 (1.7)				
**PHR**	*n* = 6, ave. sim. = 40.37		*n* = 6, ave. sim. = 28.77		*n* = 3, ave. sim. = 17.47	
	*Microdictyon setchellianum*	34.9 (15.8)	turf algae	26.7 (8.2)	*Microdictyon setchellianum*	25.9 (21.1)
	turf algae	31.0 (9.6)	*Microdictyon setchellianum*	21.0 (8.6)	turf algae	20.8 (8.8)
	*Halimeda velasquezii*	8.1 (3.1)	*Halimeda distorta*	8.7 (8.7)	*Porites compressa*	20.5 (20.5)
	*Porites lobata*	5.8 (3.1)	*Halimeda opuntia*	7.1 (7.1)	Sand	12.9 (8.4)
	*Lobophora variegata*	5.0 (1.5)	Sand	4.7 (2.1)	*Montipora capitata*	12.0 (12.0)
	*Pocillopora ligulata*	2.1 (1.7)	*Montipora capitata*	4.5 (4.5)	*Stypopodium flabelliforme*	3.5 (3.5)
**Midway**	*n* = 4, ave. sim. = 42.20		*n* = 3, ave. sim. = 60.40		*n* = 2, ave. sim. = 20.80	
	turf algae	48.6 (12.3)	turf algae	37.2 (1.6)	turf algae	51.4 (33.4)
	*Dictyota ceylanica*	10.9 (8.0)	*Montipora flabellata*	25.6 (12.8)	*Microdictyon setchellianum*	15.8 (15.8)
	Sand	10.9 (9.1)	*Laurencia galtsoffii*	13.2 (11.1)	Cyanobacteria	12.6 (12.6)
	*Laurencia galtsoffii*	6.9 (6.9)	Sand	4.4 (1.4)	*Padina* sp.	2.6 (2.6)
	*Microdictyon setchellianum*	6.3 (6.3)	*Montipora turgescens*	4.3 (2.3)	CCA	2.4 (0.8)
	*Lobophora variegata*	5.8 (2.3)	CCA	3.9 (1.3)	*Pavona varians*	2.4 (2.4)
					*Stypopodium flabelliforme*	2.4 (2.4)
**Kure**	*n* = 3, ave. sim. = 67.73		*n* = 4, ave. sim. = 66.00		*n* = 2, ave. sim. = 56.40	
	*Microdictyon setchellianum*	26.3 (2.0)	turf algae	46.1 (6.7)	*Boodlea composita*	36.8 (11.2)
	turf algae	25.5 (7.9)	*Microdictyon setchellianum*	18.2 (3.4)	turf algae	21.2 (3.2)
	*Pocillopora meandrina*	15.6 (3.7)	*Sargassum* sp.	7.1 (7.0)	*Microdictyon setchellianum*	19.6 (10.8)
	*Lobophora variegata*	8.1 (1.2)	CCA	5.3 (0.8)	*Porites compressa*	7.6 (7.6)
	*Stypopodium flabelliforme*	5.1 (5.1)	Sand	5.0 (2.1)	Sand	4.4 (0.8)
	CCA	4.8 (1.8)	*Laurencia galtsoffii*	4.3 (3.5)	CCA	3.4 (3.0)

Average percent cover is followed by standard error in parentheses. “*n*” = the number of sites surveyed in each island/habitat, “ave. sim.” = average similarity (as determined through SIMPER analyses) of sites within each island/habitat.

Most backreef sites in atoll systems also harbored similar types of communities, with all containing turf algal populations (26.7%–70.4%) and patches of sand (0.8%–5.0%) (Table 2). However, a percent cover of ∼45% for species of *Montipora* in backreef regions at Midway Atoll (an organism almost completely lacking from surveyed backreefs at Kure Atoll) coupled with a lack of *M. setchellianum* at sites in backreefs at Midway Atoll (an organism that exhibited a percent cover of 14.4%–21.0% in backreef sites at all other atolls) helped cause significant differences in the MID to KUR comparison. Although sites from lagoon systems at all 4 atolls were statistically similar, SIMPER found the scleractinian coral species *Acropora cytherea* and *Porites lobata* to be common at surveyed sites in the lagoon at French Frigate Shoals, but less prevalent at sites in the lagoons of the three northernmost atolls where *Porites compressa* and *Montipora capitata* become more common (Table 2).

Benthic cover at sites within both FFS and MID were statistically similar regardless of habitat (e.g. FFS and MID) (Table 2). For instance, all sites at FFS contained a high percent cover of turf algae (28.9%–70.4%) mixed with *Lobophora variegata* (1.2%–10.7%), while all MID sites were characterized by similar dense turf algal populations (37.2%–51.4%). At PHR and KUR, benthic cover differed significantly among surveyed sites from some or all habitat types (Table 2). At PHR, SIMPER found that the corals *Porites compressa* and *Montipora capitata* covered 20.5% and 12.0% of substrata in lagoonal regions, whereas they were absent at surveyed sites in forereef regions (Table 2). Conversely, algae such as *Halimeda velasquezii* and *Lobophora variegata*, which covered 8.1% and 5.0% of forereef regions, respectively, were not common components of surveyed lagoonal sites.

Intra-island benthic cover at forereef sites was similar within each island surveyed. As an example, all FFS forereef sites contained benthic communities with turf algae (24.4%–67.2%), *Lobophora variegata* (3.2%–23.6%), *Porites lobata* (1.2%–22.0%), and *Pocillopora meandrina* (0.4%–15.6%) (Table S5), although northern and eastern sites also were found to contain a considerable amount of *Halimeda velasquezii* (3.2%–12.0%).

**Figure 3:**
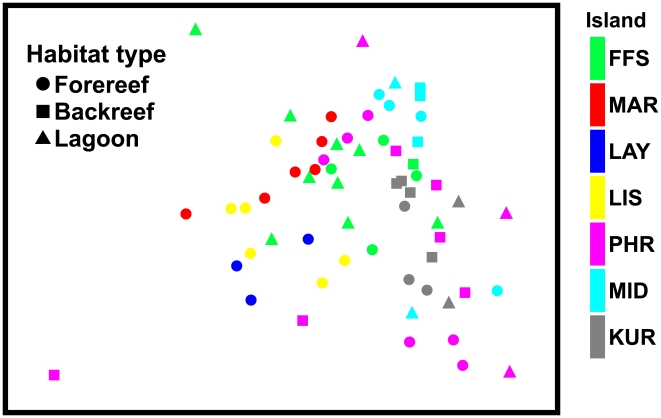
nMDS ordination depicting relationship of sites surveyed. Relationships are based on percent cover of benthic organisms (2D stress = 0.19). Tick marks on the x-axis visually separate data from each island.

A non-metric multidimensional scaling ordination (nMDS) revealed an amorphous cluster of sites when single factors (habitat, quadrant, island) were plotted. However, when both island and habitat factors were shown (Fig. 3), some structure became visible. For example, benthic communities in forereef sites at Laysan Island (blue circles), Maro Reef (red circles), and Kure Atoll (gray circles) formed clusters that were distinct from each other, indicating differences in species composition and percent cover among these communities. Alternatively, as indicated by PERMANOVA tests, many forereef sites from atoll systems overlapped (FFS, PHR, MID, KUR), indicating similarity.

## Discussion

The Northwestern Hawaiian Islands represent minimally impacted subtropical reef ecosystems that receive both federal and state protection [1] and are dominated by algal functional groups [2], [38], [48]. Such algal functional groups are vitally important for healthy reef function [2] and, contrary to currently accepted models of reef health [49], historical reports [50]–[52] indicate many near-pristine, Hawaiian, subtropical reef systems naturally contain expansive populations of macroalgae, with scleractinian coral species usually only being dense in localized areas. As such, the modern ecological data collected during this study can be accepted at face value as representing healthy reef attributes, and high abundances of macroalgae cannot simply be excused as representing impacted ecosystems suffering from unknown anthropogenic interferences.

Few species-level, interdisciplinary baselines have yet been published for unimpacted Pacific reef systems; however, in the equatorial Line Islands, coral cover is reported to range from 15% to 44% (macroalgal cover ranges not provided) [4], while in the US Phoenix Islands, coral and macroalgal covers are reported to range from 5% to 43% and 9% to 21%, respectively [3]. In the more anthropogenically impacted islands of American Samoa, island-wide averages of mean live coral cover are reported to range from 14.5% to 60.0% and mean macroalgal cover is reported to range from 6% to 36% [53]. In subtropical environments, reports of high macroalgal abundance similar to those found in the NWHI exist for high latitude reef systems in Australia [54] and suggest cooler water temperatures [10] may limit the number of coral species that can thrive in such environments while allowing macroalgal assemblages to flourish. This does not negate the ecological importance of reef building species to these ecosystems, but raises the question about the relative percent cover of coral, crustose coralline red algae, and calcified macroalgae that a healthy reef system must contain for reef accretion to occur [55]. The attributes of one “healthy” reef may not necessarily be the same as another healthy reef, and only by gaining baseline understanding of each individual reef system can we decide how best to implement management strategies.

In 2008, percent cover of coral assemblages in the NWHI varied dramatically among sites depending on which islands or habitats were considered; however, 76% of sites surveyed in 2008 contained coral cover equal to or greater than coral cover reported for these generalized geographic areas in past reef assessments (1,29–34). Unlike coral populations, tropical-to-subtropical algal assemblages change rapidly and tend to fluctuate seasonally or within and between sampling years [37], [56], and caution must be used when comparing algal percent cover data from different temporal periods. However, percent cover of macroalgal assemblages reported here is in-line with previously published reports from the NWHI [2], [5], [37]. Forereef areas at French Frigate Shoals and Pearl and Hermes Atoll [2], [36], [48] contained expansive meadows of the green macroalga *Microdictyon setchellianum*, and lagoonal areas of all atolls supported localized high densities of species of *Halimeda* (Table S2). The lack of *Microdictyon* at sites surveyed at Maro Reef, Laysan Island, and Lisianski Island during the 2008 surveys was unexpected since this alga has been reported from Gardner Pinnacles (another non-atoll island in the NWHI) [37]. Species of the brown macroalgal genera *Padina*, *Sargassum*, and *Stypopodium* became increasingly important in terms of percent cover in higher latitude reef systems (Table S2) [1], [38], likely because of cold water influences [57].

Similar to the main Hawaiian Islands [46], [58], [59], wave exposure has been observed to shape benthic communities in the PMHM [1], [9], [10]. Therefore, it was expected that reefs from quadrants experiencing dissimilar wave energy in this study would contain biologically distinct benthic communities. However, contrary to the findings of Kenyon et al. [29]–[34] who reported coral assemblages to differ depending on orientation to prevailing wave exposure, the diversity and abundance of common benthic species found in each habitat type did not differ significantly among the quadrants surveyed in this study (Table S1). This surprising outcome may occur because (1) the towed-diver surveys analyzed by Kenyon et al. [29]–[34] represented a much larger geographic scale, and the discrete REA surveys discussed here may not be able to adequately detect slight nuances of community structure between geographic area within a single habitat type, or (2) although coral community structure may differ among quandrants, when entire benthic communities (where algae often cover a significantly greater percentage of substrate than coral assemblages) are compared at the species-level, overall benthic composition between quadrants may not differ significantly.

In addition to wave exposure, tropical upwelling events and internal waves shape benthic communities and have been linked to naturally high occurrences of tropical reef macroalgae [60], [61]. Sub-surface water temperature recorders deployed during the course of this study indicate that large upwelling events wash over fore-reef areas on atoll systems in the NWHI, and the location of these upwelling events anecdotally corresponds to the location of dense beds of the green alga *Microdictyon* (CRED, unpublished data). Ongoing analyses will determine if a link exists between these upwelling events and the *Microdictyon* meadows that cover kilometers of forereef area in the NWHI.

Herbivory also strongly influences algal assemblages in tropical reef systems [62]–[64]. In the Caribbean, a die-off of *Diadema* fostered coral to macroalgal phase shifts in environments already heavily impacted by anthropogenic activities [65]. In the NWHI where human activities are limited, native urchins are a common component of the majority of sites visited. However, their abundances were low enough that they were not reported along transects at 70% of sites surveyed (although they were likely present at most sites), and their overall cover across all 62 sites surveyed was only 0.3% (SE 0.1; range 0–2.0%) (Table S4). No correlation was found between high fleshy macroalgal cover and urchin abundance (data not shown), and it seems unlikely that a lack of urchins is responsible for algal meadow formation at forereef sites in atoll systems. Because dense and diverse herbivorous fish communities were never the target of past fishing industries in the NWHI [66], fish abundances remain high along the entire NWHI chain. Species of *Kyphosus* (chubs) and the spectra-clad parrotfish (*Chlorurus perspicillatus*) represent 49% of herbivore fish biomass [6], but no correlation between these species and high fleshy macroalgal cover was found (data not shown).

This study characterizes benthic communities at islands along the NWHI chain and highlights similarities and differences in benthic composition among habitat types and geographic locations. Although this percent cover data provides insight into the most dominant organisms that occurred in each location (Tables S2, S3, S4), these sites were typically located at ∼13-m depths and only serve as representatives for the habitats and islands they represent. It is clear that each site is only a snapshot of the numerous benthic communities that occur along the NWHI chain, and assumptions cannot be made that they are representative of all existent depths and environments. However, because of the remote location, logistical constraints, and expense of sampling associated with monitoring in the NWHI, the data presented here offer a quantitative glimpse of the most common organisms present in many reef communities and form a critical baseline for future monitoring. Similarly, although the biodiversity reported in this study reflects dominant members of each site surveyed, many additional coral and algal species were anecdotally observed to occur off of survey lines, and the data presented here cannot be assumed to provide an approximation of overall diversity [see 28,37,48 for more complete biodiversity analyses].

Obviously, a high degree of biological heterogeneity exists in benthic communities within single islands and across all the islands in the NWHI Archipelago. No simple metric will ever be able to adequately predict the types of organisms that should occur on a particular reef and no “blanket value” of percent cover can serve as a universal indicator that will allow scientists or managers to determine whether or not a reef system is healthy. Instead, the initial assessments of individual reefs or islands presented here, followed by persistent monitoring, are essential for providing the numerous metrics necessary to determine whether a reef is changing over time and whether a reef can be labeled “at risk.” In the “near-pristine” NWHI [11], low coral cover and high macroalgal cover are normal for many habitat types, and scientists must educate the public that stereotypical views of healthy reef systems with coral-dominated environments do not reflect the reality of many unimpacted, and still healthy, reef environments. The data presented here and in other recent manuscripts [1]–[5], [29]–[34], [40], [42] provide a first step in reaching this goal, and will serve as the baseline for future researchers.

## Supporting Information

Table S1 Metadata for 62 sites sampled during 2008 baseline surveys.
(0.15 MB DOC)

Table S2 Percent cover of macroalgal species by site. Metadata for each site is presented in Table S1. Sum totals for each row equal the percent cover of macroalgae recorded in Table S4.
(0.48 MB DOC)

Table S3 Percent cover of scleractinian coral species by site. Metadata for each site is presented in Table S1. Sum totals for each row equal the percent cover of coral recorded in Table S4.
(0.30 MB DOC)

Table S4 Percent cover of benthic functional groups at sites in the Northwestern Hawaiian Islands. Metadata for each site is presented in Table S1. Percent cover of macroalgal and scleractinian coral species are presented in Tables S2 and S3.
(0.15 MB DOC)

Table S5 Average percent cover of the six most dominant items occurring at forereefs on each island by Quadrant (NW, NE, SE, SW). Average percent cover is followed by standard error in parentheses. “n” = the number of sites surveyed in each island/quadrant, “ave. sim.” = average similarity (as determined through SIMPER analyses) of sites within each island/quadrant.
(0.08 MB DOC)
