# Supplementary material for: Benthic Composition of a Healthy Subtropical Reef: Baseline Species-Level Cover, with an Emphasis on Algae, in the Northwestern Hawaiian Islands
Source: PLoS One. 2010 Mar 17;5(3):e9733. doi: 10.1371/journal.pone.0009733 (PMC2840019; doi:10.1371/journal.pone.0009733)
Supplement: Table S1 — Metadata for 62 sites sampled during 2008 baseline surveys. (0.15 MB DOC) [file pone.0009733.s001.doc]

| **Island** | **Site** | **Habitat** | **Quadrant** | **Depth (m)** | **Latitude** | **Longitude** | **Date visited** |
| --- | --- | --- | --- | --- | --- | --- | --- |
| **FFS** | FFS-H6 | Forereef | NW | 8.5–10.6 | 23°52.806 N | 166°16.384 W | 15 Sept 2008 |
|  | FFS-12 | Lagoon | SE | 9.4–10.1 | 23°38.301 N | 166°10.803 W | 16 Sept 2008 |
|  | FFS-21 | Lagoon | NW | 7.0–10.5 | 23°50.809 N | 166°19.612 W | 15 Sept 2008 |
|  | FFS-22 | Lagoon | NW | 2.7–3.4 | 23°51.954 N | 166°15.324 W | 8 Oct 2008 |
|  | FFS-25 | Forereef | SE | 9.2–11.0 | 23°42.249 N | 166°03.648 W | 16 Sept 2008 |
|  | FFS-29 | Forereef | NE | 8.8–10.1 | 23°50.613 N | 166°08.204 W | 8 Oct 2008 |
|  | FFS-R29 | Lagoon | SE | 9.0–15.0 | 23°40.697 N | 166°08.799 W | 16 Sept 2008 |
|  | FFS-30 | Lagoon | NW | 8.5–10.5 | 23°50.986 N | 166°17.868 W | 15 Sept 2008 |
|  | FFS-R30 | Backreef | NW | 0.6–0.9 | 23°51.522 N | 166°12.354 W | 9 Oct 2008 |
|  | FFS-32 | Lagoon | NW | 4.9–9.8 | 23°48.366 N | 166°13.838 W | 9 Oct 2008 |
|  | FFS-33 | Lagoon | NW | 8.8–11.0 | 23°50.188 N | 166°16.010 W | 8 Oct 2008 |
|  | FFS-34 | Forereef | SE | 9.5–10.0 | 23°37.675 N | 166°08.123 W | 14 Sept 2008 |
|  | FFS-35 | Lagoon | NW | 13.0–16.2 | 23°47.437 N | 166°13.921 W | 7 Oct 2008 |
|  | FFS-R46 | Lagoon | NW | 6.4–10.7 | 23°46.157 N | 166°15.704 W | 9 Oct 2008 |
| **Maro Reef** | MAR-R1 | Forereef | NW | 8.5­–10.5 | 25°27.678 N | 170°40.524 W | 18 Sept 2008 |
|  | MAR-R3 | Forereef | NW | 12.2–17.4 | 25°25.131 N | 170°40.167 W | 18 Sept 2008 |
|  | MAR-08 | Forereef | NW | 2.7–7.0 | 25°24.996 N | 170°35.025 W | 19 Sept 2008 |
|  | MAR-R12 | Forereef | NW | 13.5­–15.5 | 25°28.267 N | 170°38.576 W | 18 Sept 2008 |
|  | MAR-22 | Forereef | SE | 14.3–18 | 25°22.726 N | 170°34.042 W | 19 Sept 2008 |
|  | MAR-32 | Forereef | SE | 11.0–14.9 | 25°21.245 N | 170°32.358 W | 19 Sept 2008 |
| **Laysan** | LAY-05 | Forereef | NE | 5.8–11.2 | 25°47.244 N | 171°43.743 W | 20 Sept 2008 |
|  | LAY-R9 | Forereef | SW | 6.0–13.7 | 25°45.238 N | 171°44.463 W | 20 Sept 2008 |
|  | LAY-R12 | Forereef | NW | 7.0–13.6 | 25°46.662 N | 171°44.833 W | 20 Sept 2008 |
| **Lisianski** | LIS-R7 | Forereef | SW | 9.7–14.0 | 25°57.237 N | 173°58.228 W | 5 Oct 2008 |
|  | LIS-10 | Forereef | SE | 8.5–11.3 | 25°56.460 N | 173°55.344 W | 5 Oct 2008 |
|  | LIS-R10 | Forereef | SW | 10.0–14.6 | 25°56.671 N | 173°57.210 W | 5 Oct 2008 |
|  | LIS-12 | Forereef | NW | 7.0–8.2 | 26°03.954 N | 174°00.102 W | 4 Oct 2008 |
|  | LIS-R14 | Forereef | NW | 13.0–14.9 | 26°04.702 N | 173°59.821 W | 4 Oct 2008 |
|  | LIS-18 | Forereef | NW | 6.4–8.8 | 26°00.257 N | 173°59.642 W | 4 Oct 2008 |
| **PHR** | PHR-22 | Backreef | SW | 1.0–1.5 | 27°47.715 N | 175°51.997 W | 2 Oct 2008 |
|  | PHR-23 | Backreef | NW | 5.2–6.4 | 27°52.868 N | 175°55.967 W | 3 Oct 2008 |
|  | PHR-24 | Backreef | NW | 4.5–6.4 | 27°55.180 N | 175°51.688 W | 23 Sept 2008 |
|  | PHR-26 | Backreef | NE | 1.5–2.1 | 27°57.479 N | 175°48.131 W | 3 Oct 2008 |
|  | PHR-R26 | Forereef | SE | 12.0–14.6 | 27°47.143 N | 175°46.825 W | 24 Sept 2008 |
|  | PHR-30 | Backreef | SW | 2.0–3.0 | 27°46.722 N | 175°53.705 W | 2 Oct 2008 |
|  | PHR-31 | Lagoon | SW | 6.4–9.1 | 27°46.532 N | 175°58.401 W | 22 Sept 2008 |
|  | PHR-R31 | Lagoon | SE | 10.7–16.0 | 27°49.586 N | 175°47.484 W | 24 Sept 2008 |
|  | PHR-32 | Lagoon | SW | 4.8–6.7 | 27°46.346 N | 175°56.376 W | 2 Oct 2008 |
|  | PHR-R32 | Backreef | SE | 0.9–1.2 | 27°50.072 N | 175°45.210 W | 24 Sept 2008 |
|  | PHR-33 | Forereef | SE | 10.6–12.7 | 27°47.128 N | 175°49.425 W | 2 Oct 2008 |
|  | PHR-34 | Forereef | SW | 10.7–18.6 | 27°45.353 N | 175°57.707 W | 22 Sept 2008 |
|  | PHR-R39 | Forereef | NW | 13.3–14.8 | 27°56.446 N | 175°51.705 W | 23 Sept 2008 |
|  | PHR-R42 | Forereef | SW | 11.3–15.5 | 27°45.188 N | 175°56.926 W | 22 Sept 2008 |
|  | PHR-R44 | Forereef | NW | 11.0–14.0 | 27°54.627 N | 175°54.276 W | 23 Sept 2008 |
| **Midway** | MID-01 | Backreef | NW | 0.2–1.2 | 28°16.155 N | 177°23.168 W | 26 Sept 2008 |
|  | MID-02 | Forereef | SE | 12.0–13.5 | 28°11.840 N | 177°20.746 W | 28 Sept 2008 |
|  | MID-03 | Lagoon | SE | 4.6–9.1 | 28°13.074 N | 177°20.644 W | 28 Sept 2008 |
|  | MID-R3 | Forereef | SW | 14.0–17.6 | 28°11.415 N | 177°23.993 W | 25 Sept 2008 |
|  | MID-R7 | Forereef | SW | 13.0–14.8 | 28°11.782 N | 177°22.501 W | 25 Sept 2008 |
|  | MID-H10 | Forereef | SW | 7.6–11.3 | 28°12.908 N | 177°25.504 W | 25 Sept 2008 |
|  | MID-H11 | Lagoon | SW | 3.0–5.5 | 28°13.060 N | 177°24.193 W | 26 Sept 2008 |
|  | MID-R20 | Backreef | SE | 0.9–2.0 | 28°13.890 N | 177°19.091 W | 28 Sept 2008 |
|  | MID-H21 | Backreef | NW | 0.9–1.2 | 28°13.060 N | 177°24.193 W | 26 Sept 2008 |
| **Kure** | KUR-02 | Forereef | NW | 12.0–13.0 | 28°27.218 N | 178°20.641 W | 30 Sept 2008 |
|  | KUR-09 | Lagoon | SW | 3.4–6.7 | 28°24.352 N | 178°20.536 W | 1 Oct 2008 |
|  | KUR-12 | Forereef | SE | 9.0–12.5 | 28°22.940 N | 178°19.474 W | 30 Sept 2008 |
|  | KUR-14 | Backreef | NW | 0.9–1.5 | 28°27.209 N | 178°19.716 W | 1 Oct 2008 |
|  | KUR-17 | Backreef | NW | 0.9–3.6 | 28°25.913 N | 178°22.004 W | 1 Oct 2008 |
|  | KUR-18 | Lagoon | NW | 3.7 –8.8 | 28°25.120 N | 178°20.675 W | 29 Sept 2008 |
|  | KUR-R33 | Forereef | SW | 13.6–15.9 | 28°25.013 N | 178°22.709 W | 29 Sept 2008 |
|  | KUR-R35 | Backreef | SW | 3.6–4.5 | 28°23.588 N | 178°20.960 W | 30 Sept 2008 |
|  | KUR-R36 | Backreef | NW | 1.8–3.9 | 28°25.221 N | 178°22.285 W | 29 Sept 2008 |

Table S1: Metadata for 62 sites sampled during 2008 baseline surveys.
