# Supplementary material for: Benthic Composition of a Healthy Subtropical Reef: Baseline Species-Level Cover, with an Emphasis on Algae, in the Northwestern Hawaiian Islands
Source: PLoS One. 2010 Mar 17;5(3):e9733. doi: 10.1371/journal.pone.0009733 (PMC2840019; doi:10.1371/journal.pone.0009733)
Supplement: Table S2 — Percent cover of macroalgal species by site. Metadata for each site is presented in Table S1. Sum totals for each row equal the percent cover of macroalgae recorded in Table S4. (0.48 MB DOC) [file pone.0009733.s002.doc]

|  |  |  | Chlorophyta | | | | | | | | | | | | | | | | | | Ochrophyta | | | | | | | | | Rhodophyta | | | | | | | | | | | | | |
| --- | --- | --- | --- | --- | --- | --- | --- | --- | --- | --- | --- | --- | --- | --- | --- | --- | --- | --- | --- | --- | --- | --- | --- | --- | --- | --- | --- | --- | --- | --- | --- | --- | --- | --- | --- | --- | --- | --- | --- | --- | --- | --- | --- |
| Island | Site | TOTAL PERCENT COVER | *Boodlea composita* | *Bryopsis pennata* | *Caulerpa racemosa* | *Caulerpa seerrulata* | *Caulerpa taxifolia* | *Caulerpa webbiana* | *Caulerpella ambigua* | *Codium arabicum* | *Codium edule* | *Dictyosphaeria cavernosa* | *Dictyosphaeria versluysii* | *Halimeda discoidea* | *Halimeda distorta* | *Halimeda gracilis* | *Halimeda opuntia* | *Halimeda velasquezii* | *Microdictyon setchellianum* | *Neomeris* spp. | *Dictyota ceylanica* | *Dictyota friabilis* | *Dictyota sandvichensis* | *Distromium flabellatum* | *Lobophora variegata* | *Padina* sp. | *Sargassum* sp. | *Stypopodium flabelliforme* | *Turbinaria ornata* | *Asparagopsis taxiformis* | *Dasya iridescens* | *Galaxaura* sp. | *Ganonema farinosum* | gelid | *Haloplegma duperreyi* | *Jania* sp. | *Kallymenia thompsonii* | *Laurencia galtsoffii* | *Laurencia majuscula* | *Liagora* sp. | *Martensia* sp. | *Peyssonnelia* sp. | *Portieria hornemannnii* |
| FFS | FFS-H6 | 22.4 | - | - | - | - | - | - | - | - | - | - | - | 2.8 | - | - | - | 12.0 | 3.2 | 0.4 | - | - | - | - | 3.2 | - | - | - | - | - | - | - | - | - | 0.8 | - | - | - | - | - | - | - | - |
|  | FFS-12 | 0.8 | - | - | - | - | - | - | - | - | - | - | - | - | - | - | - | - | - | 0.4 | - | - | - | - | 0.4 | - | - | - | - | - | - | - | - | - | - | - | - | - | - | - | - | - | - |
|  | FFS-21 | 6.4 | - | - | - | - | - | - | - | - | - | - | - | 1.6 | - | - | - | 4.4 | - | - | - | - | - | - | 0.4 | - | - | - | - | - | - | - | - | - | - | - | - | - | - | - | - | - | - |
|  | FFS-22 | 36.8 | - | - | - | - | - | - | - | - | - | - | 0.4 | - | - | - | - | - | 22.8 | - | - | - | - | - | 12.0 | - | - | - | - | - | - | - | - | 0.4 | - | - | - | - | 0.4 | 0.8 | - | - | - |
|  | FFS-25 | 53.2 | - | - | - | - | - | - | - | - | - | - | - | 3.2 | - | - | - | 12.0 | 30.0 | 0.4 | - | - | - | - | 7.6 | - | - | - | - | - | - | - | - | - | - | - | - | - | - | - | - | - | - |
|  | FFS-29 | 28.4 | - | - | - | - | - | - | - | - | - | - | - | - | - | - | - | 3.2 | 1.6 | - | - | - | - | - | 23.6 | - | - | - | - | - | - | - | - | - | - | - | - | - | - | - | - | - | - |
|  | FFS-R29 | 14.4 | - | - | - | - | - | - | - | - | - | - | 0.4 | 8.0 | - | - | 0.4 | 3.2 | - | - | - | - | - | - | 2.4 | - | - | - | - | - | - | - | - | - | - | - | - | - | - | - | - | - | - |
|  | FFS-30 | 31.6 | - | - | - | - | - | - | - | - | - | - | - | 2.0 | - | - | - | 27.6 | - | - | - | - | - | - | 1.6 | - | - | - | - | - | - | - | - | - | - | - | - | - | 0.4 | - | - | - | - |
|  | FFS-R30 | 20.0 | - | - | - | - | - | - | - | - | - | 0.4 | 0.4 | - | - | - | - | - | 14.4 | - | - | - | - | - | 1.2 | - | - | - | - | - | - | - | - | 0.8 | - | 2.4 | - | - | - | 0.4 | - | - | - |
|  | FFS-32 | 44.8 | - | - | - | 0.4 | - | - | - | - | - | - | - | 0.4 | 2.0 | - | 0.4 | 9.6 | 13.2 | - | - | - | - | - | 18.8 | - | - | - | - | - | - | - | - | - | - | - | - | - | - | - | - | - | - |
|  | FFS-33 | 29.2 | - | - | - | - | - | - | - | - | - | - | - | - | - | 0.8 | - | 8.0 | 0.4 | - | - | - | - | - | 20.0 | - | - | - | - | - | - | - | - | - | - | - | - | - | - | - | - | - | - |
|  | FFS-34 | 25.6 | - | - | - | - | - | - | - | - | - | - | - | 0.4 | - | - | - | 0.4 | 16.4 | - | - | - | - | - | 8.4 | - | - | - | - | - | - | - | - | - | - | - | - | - | - | - | - | - | - |
|  | FFS-35 | 25.2 | - | - | - | - | - | - | - | 0.8 | - | - | - | - | - | - | 2.4 | 12.8 | 2.4 | - | - | - | - | - | 5.2 | - | - | - | - | - | - | - | - | - | - | - | - | - | 1.6 | - | - | - | - |
|  | FFS-R46 | 10.4 |  |  |  |  |  |  |  |  |  |  |  |  |  |  |  |  |  |  |  | 1.6 |  |  | 6.0 |  |  |  |  | 2.8 |  |  |  |  |  |  |  |  |  |  |  |  |  |
| Maro Reef | MAR-R1 | 14.8 | - | - | - | - | 0.4 | - | - | - | - | - | - | 0.4 | - | - | - | 10.8 | - | - | - | - | - | - | - | - | - | - | - | - | - | - | - | - | - | - | - | - | 3.2 | - | - | - | - |
|  | MAR-R3 | 8.8 | - | 0.8 | - | - | - | - | - | - | - | - | - | - | - | - | - | 7.2 | - | - | - | - | - | - | 0.8 | - | - | - | - | - | - | - | - | - | - | - | - | - | - | - | - | - | - |
|  | MAR-08 | 14.4 | - | 0.4 | - | - | - | - | - | - | - | - | - | - | - | - | - | 14.0 | - | - | - | - | - | - | - | - | - | - | - | - | - | - | - | - | - | - | - | - | - | - | - | - | - |
|  | MAR-R12 | 32.4 | - | 9.2 | 0.4 | - | - | - | - | - | - | - | - | 1.6 | - | - | 21.2 | - | - | - | - | - | - | - | - | - | - | - | - | - | - | - | - | - | - | - | - | - | - | - | - | - | - |
|  | MAR-22 | 8.4 | - | - | - | - | - | - | - | - | - | - | - | 1.2 | - | - | 0.8 | 6.4 | - | - | - | - | - | - | - | - | - | - | - | - | - | - | - | - | - | - | - | - | - | - | - | - | - |
|  | MAR-32 | 17.2 | - | - | - | - | - | - | - | - | - | - | - | - | - | - | 13.2 | 3.6 | - | - | - | - | - | - | 0.4 | - | - | - | - | - | - | - | - | - | - | - | - | - | - | - | - | - | - |
| LAY | LAY-05 | 54.0 | - | - | - | - | - | - | - | - | - | - | - | - | - | - | - | 44.0 | - | - | 0.4 | - | - | - | 0.4 | - | - | - | - | - | - | - | - | - | - | - | - | - | 8.4 | - | - | 0.8 | - |
|  | LAY-R9 | 49.2 | - | - | - | - | - | - | - | - | - | - | - | - | - | - | - | 32.8 | 0.4 | - | 3.6 | - | - | - | - | - | - | - | - | - | - | - | - | - | - | - | - | - | 11.2 | - | - | 0.4 | 0.8 |
|  | LAY-R12 | 51.2 | - | - | - | - | - | 0.4 | - | - | - | - | - | 0.4 | - | - | - | 37.6 | 6.4 | - | 0.4 | - | - | - | 0.8 | - | - | - | - | - | - | 0.4 | - | - | 0.4 | - | - | - | 2.8 | - | - | 1.2 | 0.4 |
| Lisianski | LIS-R7 | 18.0 | - | - | - | - | - | - | 0.4 | - | - | 6.0 | 1.2 | - | - | - | 2.4 | 3.2 | 2.8 | 0.4 | - | - | - | - | 0.8 | - | - | - | - | - | - | - | - | 0.8 | - | - | - | - | - | - | - | - | - |
|  | LIS-10 | 2.8 | - | - | - | - | - | - | - | - | - | - | 0.4 | 0.4 | - | - | - | 0.8 | - | - | - | - | - | - | 1.2 | - | - | - | - | - | - | - | - | - | - | - | - | - | - | - | - | - | - |
|  | LIS-R10 | 17.6 | - | - | - | - | - | - | - | - | - | - | - | - | - | - | 5.6 | 5.2 | - | - | - | - | - | - | 6.4 | - | - | - | - | - | - | - | - | - | - | - | - | - | - | - | - | 0.4 | - |
|  | LIS-12 | 48.4 | - | - | - | - | - | - | - | - | - | 0.4 | 1.2 | - | - | - | 24.0 | 10.4 | 11.2 | - | - | - | - | - | 0.8 | - | - | - | - | - | - | - | - | - | - | - | - | - | - | - | - | 0.4 | - |
|  | LIS-R14 | 29.2 | - | - | - | - | - | - | - | - | - | - | - | - | - | - | 11.2 | 10.0 | 3.2 | - | - | - | - | - | 4.8 | - | - | - | - | - | - | - | - | - | - | - | - | - | - | - | - | - | - |
|  | LIS-18 | 54.4 | - | - | - | - | - | - | - | - | - | - | 2.8 | - | - | - | 13.6 | 10.0 | 20.8 | - | - | - | - | - | 5.2 | - | - | - | - | - | - | - | - | - | - | - | - | 1.2 | - | - | 0.8 | - | - |
| PHR | PHR-22 | 64.4 | - | - | - | - | - | - | - | - | - | - | 2.8 | - | - | - | - | - | 58.0 | - | - | - | - | - | - | - | - | - | - | - | - | - | 3.6 | - | - | - | - | - | - | - | - | - | - |
|  | PHR-23 | 64.0 | - | - | - | - | - | 0.8 | - | 2.0 | 0.4 | 0.4 | 0.8 | - | - | - | 42.4 | 4.0 | 8.8 | - | 0.4 | - | - | - | 1.2 | - | - | - | 0.8 | - | - | - | - | 0.4 | - | - | 0.4 | - | - | - | - | 1.2 | - |
|  | PHR-24 | 64.4 | - | - | - | - | - | - | - | - | - | - | 0.8 | 6.4 | 52.4 | - | - | 4.4 | - | - | - | - | - | - | - | - | - | - | - | - | 0.4 | - | - | - | - | - | - | - | - | - | - | - | - |
|  | PHR-26 | 21.6 | - | - | - | - | - | - | - | 0.4 | - | 1.6 | 0.8 | 1.6 | - | - | - | 1.6 | 8.4 | - | - | - | - | - | - | - | - | - | - | - | - | - | - | - | - | - | - | 7.2 | - | - | - | - | - |
|  | PHR-R26 | 72.4 | - | - | - | - | - | - | - | - | - | - | 0.4 | 0.4 | - | - | - | 7.6 | 58.4 | 0.4 | - | - | - | - | 1.6 | 0.8 | - | - | - | - | - | - | 0.4 | - | - | - | - | 2.4 | - | - | - | - | - |
|  | PHR-30 | 58.4 | - | - | - | - | - | - | - | - | - | - | 0.4 | - | - | - | - | - | 30.0 | - | - | - | - | - | 4.8 | - | - | 0.4 | - | - | - | - | 5.6 | - | - | - | - | 17.2 | - | - | - | - | - |
|  | PHR-31 | 14.4 | - | - | - | - | - | - | - | - | - | - | 0.8 | 1.2 | 1.6 | - | - | 0.4 | 10.0 | 0.4 | - | - | - | - | - | - | - | - | - | - | - | - | - | - | - | - | - | - | - | - | - | - | - |
|  | PHR-R31 | - | - | - | - | - | - | - | - | - | - | - | - | - | - | - | - | - | - | - | - | - | - | - | - | - | - | - | - | - | - | - | - | - | - | - | - | - | - | - | - | - | - |
|  | PHR-32 | 80.4 | - | - | - | - | - | - | - | - | - | 0.8 | - | - | - | - | - | - | 67.6 | 0.4 | - | - | - | - | - | 1.2 | - | 10.4 | - | - | - | - | - | - | - | - | - | - | - | - | - | - | - |
|  | PHR-R32 | 26.0 | - | - | - | - | - | - | - | - | - | - | - | - | - | - | - | 2.0 | 20.8 | - | - | - | - | - | - | - | - | - | - | - | - | 0.4 | 2.8 | - | - | - | - | - | - | - | - | - | - |
|  | PHR-33 | 82.4 | - | - | - | - | - | - | - | - | - | - | 0.4 | 1.2 | - | - | - | 0.4 | 76.0 | - | - | - | - | - | 3.6 | - | - | - | - | - | - | - | 0.4 | - | - | - | - | 0.4 | - | - | - | - | - |
|  | PHR-34 | 16.0 | - | - | - | - | - | - | - | - | - | - | - | - | - | - | - | 6.0 | - | - | - | - | - | - | 10.0 | - | - | - | - | - | - | - | - | - | - | - | - | - | - | - | - | - | - |
|  | PHR-R39 | 23.2 | - | - | - | - | - | - | - | - | - | - | - | - | - | - | - | 13.2 | 0.4 | 0.4 | - | - | - | - | 8.8 | - | - | - | - | - | - | - | - | - | - | - | - | - | - | - | - | - | 0.4 |
|  | PHR-R42 | 83.2 | - | - | - | - | - | - | - | - | - | - | - | 0.8 | - | - | - | 0.8 | 74.8 | 0.4 | - | - | - | - | 5.2 | 0.4 | - | - | - | - | - | - | 0.8 | - | - | - | - | - | - | - | - | - | - |
|  | PHR-R44 | 22.0 | - | - | - | - | - | - | - | - | - | - | - | 0.8 | - | - | - | 20.4 | - | - | - | - | - | - | 0.8 | - | - | - | - | - | - | - | - | - | - | - | - | - | - | - | - | - | - |
| Midway | MID-01 | 9.2 | - | - | - | - | - | - | - | - | - | - | 0.4 | - | - | - | - | - | - | - | - | - | - | - | 3.6 | - | 0.4 | - | 0.4 | - | - | - | - | - | - | - | - | 4.4 | - | - | - | - | - |
|  | MID-02 | 44.8 | - | - | - | - | - | - | - | - | - | 0.4 | - | - | - | - | - | - | 25.2 | - | 7.6 | - | - | - | 0.4 | 10.8 | - | - | - | - | 0.4 | - | - | - | - | - | - | - | - | - | - | - | - |
|  | MID-03 | 0.8 | - | - | - | - | - | - | - | - | - | - | - | - | - | - | - | - | - | - | - | - | - | - | 0.8 | - | - | - | - | - | - | - | - | - | - | - | - | - | - | - | - | - | - |
|  | MID-R3 | 12.4 | - | - | - | - | - | - | - | - | - | - | - | - | - | - | - | - | - | - | 1.2 | - | - | - | 10.8 | 0.4 | - | - | - | - | - | - | - | - | - | - | - | - | - | - | - | - | - |
|  | MID-R7 | 39.2 | - | - | - | - | - | - | - | - | - | - | - | - | - | - | - | - | - | - | 34.4 | - | - | - | 4.0 | 0.8 | - | - | - | - | - | - | - | - | - | - | - | - | - | - | - | - | - |
|  | MID-H10 | 36.8 | - | - | - | - | - | - | - | - | - | - | - | - | - | - | - | - | - | - | 0.4 | - | - | - | 8.0 | 0.8 | - | - | - | - | - | - | - | - | - | - | - | 27.6 | - | - | - | - | - |
|  | MID-H11 | 49.2 | 0.4 | - | - | - | - | - | - | 0.8 | - | - | - | - | - | - | - | 0.8 | 31.6 | - | - | - | 1.6 | - | 2.0 | 5.2 | - | 4.8 | 0.4 | - | - | 0.4 | - | - | - | - | - | 1.2 | - | - | - | - | - |
|  | MID-R20 | 48.8 | - | - | - | - | - | - | - | - | - | - | 0.4 | 1.2 | - | - | - | 0.4 | 2.4 | - | - | - | - | - | 2.4 | - | - | 6.8 | - | - | - | - | - | - | - | - | - | 35.2 | - | - | - | - | - |
|  | MID-H21 | 6.8 | - | - | - | - | - | - | - | - | - | - | - | - | - | - | - | - | - | - | - | - | - | - | 3.6 | - | 2.8 | - | - | - | - | 0.4 | - | - | - | - | - | - | - | - | - | - | - |
| Kure | KUR-02 | 46.8 | - | - | - | - | - | - | - | - | - | - | - | 0.4 | - | - | - | - | 28.8 | - | - | - | - | - | 6.0 | 1.6 | - | - | 4.0 | - | - | - | - | - | - | - | - | 6.0 | - | - | - | - | - |
|  | KUR-09 | 65.2 | 48.0 | - | - | - | - | - | - | - | - | 0.4 | - | - | - | - | - | 1.2 | 8.8 | - | - | - | - | - | 5.2 | - | - | 1.6 | - | - | - | - | - | - | - | - | - | - | - | - | - | - | - |
|  | KUR-12 | 64.4 | - | - | - | - | - | - | - | - | - | - | - | 0.8 | - | - | - | - | 27.6 | - | - | - | - | 4.8 | 10.0 | 0.4 | - | 15.2 | - | - | 0.4 | - | - | - | - | - | - | 5.2 | - | - | - | - | - |
|  | KUR-14 | 31.2 | - | - | - | - | - | - | - | - | - | - | - | - | - | - | - | - | 14.0 | - | - | - | - | - | 2.4 | - | - | - | - | - | - | - | - | - | - | - | - | 14.8 | - | - | - | - | - |
|  | KUR-17 | 21.2 | - | - | - | - | - | - | - | - | - | - | - | - | - | - | - | - | 13.2 | - | - | - | - | 0.4 | 4.4 | - | - | 0.8 | - | - | - | - | - | - | - | - | - | 2.4 | - | - | - | - | - |
|  | KUR-18 | 61.2 | 25.6 | - | - | - | - | - | - | - | - | - | 5.2 | - | - | - | - | - | 30.4 | - | - | - | - | - | - | - | - | - | - | - | - | - | - | - | - | - | - | - | - | - | - | - | - |
|  | KUR-R33 | 36.4 | - | - | - | - | - | - | - | 0.4 | - | - | - | 1.2 | - | - | - | - | 22.4 | - | 2.0 | - | - | - | 8.4 | 0.8 | 0.4 | - | 0.4 | - | - | - | - | - | - | - | - | - | - | - | - | - | 0.4 |
|  | KUR-R35 | 66.4 | 5.6 | - | - | - | - | - | - | - | - | 0.4 | - | 0.8 | - | - | - | - | 28.0 | - | 0.8 | - | - | - | 2.0 | 0.4 | 28.0 | 0.4 | - | - | - | - | - | - | - | - | - | - | - | - | - | - | - |
|  | KUR-R36 | 22.4 | - | - | - | - | - | - | - | - | - | - | - | 0.4 | - | - | - | - | 17.6 | - | - | - | - | - | 2.4 | 0.8 | 0.4 | - | 0.8 | - | - | - | - | - | - | - | - | - | - | - | - | - | - |

Table S2: Percent cover of macroalgal species by site. Metadata for each site is presented in Table S1. Sum totals for each row equal the percent cover of macroalgae recorded in Table S4.
