# Supplementary material for: Benthic Composition of a Healthy Subtropical Reef: Baseline Species-Level Cover, with an Emphasis on Algae, in the Northwestern Hawaiian Islands
Source: PLoS One. 2010 Mar 17;5(3):e9733. doi: 10.1371/journal.pone.0009733 (PMC2840019; doi:10.1371/journal.pone.0009733)
Supplement: Table S3 — Percent cover of scleractinian coral species by site. Metadata for each site is presented in Table S1. Sum totals for each row equal the percent cover of coral recorded in Table S4. (0.30 MB DOC) [file pone.0009733.s003.doc]

| Island | Site | TOTAL PERCENT COVER | *Acropora cytherea* | *Acropora humilis* | *Acropora valida* | *Cyphastrea ocellina* | *Fungia scutaria* | *Leptastrea purpurea* | *Montipora capitata* | *Montipora flabellata* | *Montipora incrassata* | *Montipora patula* | *Montipora turgescens* | *Pavona duerdeni* | *Pavona variens* | *Pocillopora damicornis* | *Pocillopora ligulata* | *Pocillopora meandrina* | *Pocillopora* sp. | *Porites brighami* | *Porites compressa* | *Porites evermanni* | *Porites lobata* | *Psammocora stellata* | *Psammocra nierstraszi* |
| --- | --- | --- | --- | --- | --- | --- | --- | --- | --- | --- | --- | --- | --- | --- | --- | --- | --- | --- | --- | --- | --- | --- | --- | --- | --- |
| French Frigate Shoals | FFS-H6 | 36.4 | 10.4 | - | - | - | - | 0.4 | 1.2 | - | - | - | - | 2.0 | - | - | - | 0.4 | - | - | - | - | 22.0 | - | - |
|  | FFS-12 | 81.6 | 80.8 | - | - | - | - | - | - | - | - | - | - | - | - | - | - | - | - | - | - | - | 0.8 | - | - |
|  | FFS-21 | 59.2 | 46.8 | 0.8 | - | - | - | - | - | - | - | - | - | 0.8 | - | - | - | 0.8 | - | - | - | - | 10.0 | - | - |
|  | FFS-22 | 1.6 | - | - | - | - | - | - | - | - | - | - | - | - | - | 0.8 | - | 0.8 | - | - | - | - | - | - | - |
|  | FFS-25 | 20.0 | - | - | - | - | - | - | - | - | - | - | - | 0.4 | - | - | - | 5.6 | - | - | - | - | 14.0 | - | - |
|  | FFS-29 | 21.6 | 1.6 | - | - | - | - | - | - | - | 0.4 | 0.8 | - | 2.0 | - | - | - | 15.6 | - | - | - | - | 1.2 | - | - |
|  | FFS-R29 | 22.4 | - | - | - | 0.8 | - | - | 0.8 | - | - | - | - | 2.4 | - | 8.8 | - | - | - | 0.4 | 7.6 | - | 1.6 | - | - |
|  | FFS-30 | 24.8 | 8.8 | 0.8 | - | - | - | - | 0.4 | - | - | - | - | 0.8 | - | - | - | 0.8 | - | - | - | - | 13.2 | - | - |
|  | FFS-R30 | 0.8 | - | - | - | - | - | - | - | - | - | - | - | - | - | 0.4 | - | - | - | - | - | - | - | 0.4 | - |
|  | FFS-32 | 19.2 | - | - | - | - | - | - | - | - | - | 2.0 | - | - | - | 0.8 | - | - | - | - | 2.4 | 3.6 | 10.4 | - | - |
|  | FFS-33 | 26.8 | - | - | - | - | - | - | 0.8 | - | - | 0.4 | - | - | - | - | - | 0.8 | - | - | 3.2 | 1.2 | 20.4 | - | - |
|  | FFS-34 | 3.6 | - | - | - | - | - | - | - | - | - | - | - | - | - | - | - | 2.0 | - | - | - | - | 1.6 | - | - |
|  | FFS-35 | 52.8 | 0.8 | - | - | - | - | - | - | - | - | - | - | - | - | - | - | - | - | - | 5.2 | 0.4 | 46.4 | - | - |
|  | FFS-R46 | 42.0 | 3.6 |  | 5.2 |  |  |  | 0.4 |  |  | 3.6 |  | 1.2 |  |  |  | 5.2 |  |  |  | 0.4 | 22.0 |  | 0.4 |
| Maro Reef | MAR-R1 | 61.6 | - | - | - | - | - | - | 15.2 | 4.8 | - | - | - | - | - | - | - | 2.4 | - | - | 19.2 | 0.8 | 19.2 | - | - |
|  | MAR-R3 | 72.8 | - | - | - | - | - | - | 23.2 | 1.6 | - | 12.0 | - | - | - | - | 2.4 | 1.6 | - | - | - | 16.8 | 15.2 | - | - |
|  | MAR-08 | 37.6 | - | - | - | - | - | - | 4.4 | - | - | - | - | - | - | - | - | 0.8 | - | - | 16.8 | - | 15.6 | - | - |
|  | MAR-R12 | 23.6 | - | - | - | - | 0.4 | - | 6.8 | - | - | - | - | - | - | - | - | - | - | - | 3.6 | 0.4 | 12.4 | - | - |
|  | MAR-22 | 32.4 | - | - | - | - | - | - | 10.8 | 1.6 | - | 3.2 | - | - | - | - | - | - | - | - | 15.2 | - | 1.6 | - | - |
|  | MAR-32 | 20.4 | - | - | - | - | - | - | - | - | - | - | - | - | - | - | - | - | - | - | 14.4 | - | 6.0 | - | - |
| LAY | LAY-05 | 7.6 | - | - | - | - | - | - | 0.4 | - | - | - | - | - | - | - | - | 7.2 | - | - | - | - | - | - | - |
|  | LAY-R9 | 24.0 | - | - | - | - | - | - | 2.4 | - | - | - | - | - | - | - | - | 0.8 | - | - | - | 0.4 | 20.4 | - | - |
|  | LAY-R12 | 12.8 | - | - | - | - | - | - | 0.4 | - | - | - | - | - | - | - | - | 2.4 | - | - | - | - | 10.0 | - | - |
| LIS | LIS-R7 | 59.2 | - | - | - | - | - | - | 4.8 | - | - | 2.4 | - | - | 0.4 | - | - | - | - | - | 8.8 | 32.8 | 10.0 | - | - |
|  | LIS-10 | 51.6 | - | - | - | - | - | - | 4.0 | - | - | 4.0 | - | - | - | - | - | - | - | - | 9.6 | 33.2 | 0.8 | - | - |
|  | LIS-R10 | 50.8 | - | - | - | 0.4 | - | - | 5.6 | - | - | 9.2 | - | - | - | - | - | - | - | - | 10.8 | 20.0 | 4.8 | - | - |
|  | LIS-12 | 5.2 | - | - | - | - | - | - | - | - | - | - | - | 0.4 | - | - | - | - | - | - | - | - | 4.8 | - | - |
|  | LIS-R14 | 45.6 | - | - | - | - | - | - | 1.2 | - | - | - | - | - | - | - | - | - | - | - | - | 14.8 | 29.6 | - | - |
|  | LIS-18 | 18.0 | - | - | - | - | - | - | - | - | - | - | - | - | - | - | - | - | - | - | - | 2.0 | 16.0 | - | - |
| Pearl and Hermes Atoll | PHR-22 | 3.2 | - | - | - | - | - | - | - | - | - | - | - | - | - | 0.8 | - | 2.4 | - | - | - | - | - | - | - |
|  | PHR-23 | 2.0 | - | - | - | - | - | - | 0.4 | - | - | - | - | - | - | - | - | - | - | - | 0.4 | - | 1.2 | - | - |
|  | PHR-24 | 8.0 | - | - | - | - | - | - |  | - | - | - | - | - | - | - | - | - | - | - | 7.6 | - | 0.4 | - | - |
|  | PHR-26 | 34.8 | - | - | - | - | - | - | 26.8 | 7.6 | - | - | 0.4 | - | - | - | - | - | - | - | - | - | - | - | - |
|  | PHR-R26 | 6.8 | - | - | - | - | - | - | - | - | - | - | - | - | - | - | - | 0.8 | - | - | - | - | 6.0 | - | - |
|  | PHR-30 | 6.0 | - | - | - | - | - | 0.8 | - | - | - | - | - | - | - | - | - | 5.2 | - | - | - | - | - | - | - |
|  | PHR-31 | 36.4 | - | - | - | - | - | 0.4 | 36.0 | - | - | - | - | - | - | - | - | - | - | - | - | - | - | - | - |
|  | PHR-R31 | 62.0 | - | - | - | - | - | - | - | - | - | - | - | - | 0.4 | - | - | - | - | - | 61.6 | - | - | - | - |
|  | PHR-32 | 3.6 | - | - | - | - | - | - | - | - | - | - | - | - | - | 0.8 | - | 2.8 | - | - | - | - | - | - | - |
|  | PHR-R32 | 0.8 | - | - | - | - | - | - | - | - | - | - | - | - | - | - | 0.4 | 0.4 | - | - | - | - | - | - | - |
|  | PHR-33 | 1.6 | - | - | - | - | - | 0.4 | - | - | - | - | - | - | - | - | - | - | - | - | - | - | 1.2 | - | - |
|  | PHR-34 | 18.4 | - | - | - | - | - | 0.4 | 0.4 | 0.4 | - | 1.2 | - | 0.4 | - | - | 10.4 | - | - | - | - | 0.4 | 4.8 | - | - |
|  | PHR-R39 | 1.6 | - | - | - | - | 0.4 | - | 0.4 | - | - | - | - | - | - | - | 0.8 | - | - | - | - | - | - | - | - |
|  | PHR-R42 | 5.2 | - | - | - | - | - | 0.8 | - | - | - | - | - | - | - | - | 1.2 | 0.4 | - | - | - | - | 2.0 | 0.8 | - |
|  | PHR-R44 | 21.2 | - | - | - | - | - | - | - | - | - | - | - | - | - | - | - | - | - | - | - | 0.4 | 20.8 | - | - |
| Midway | MID-01 | 48.4 | - | - | - | - | - | - | 1.2 | 39.2 | - | - | 8.0 | - | - | - | - | - | - | - | - | - | - | - | - |
|  | MID-02 | 1.2 | - | - | - | - | - | - | - | - | - | - | - | - | - | - | - | 0.8 | - | - | - | - | 0.4 | - | - |
|  | MID-03 | 9.6 | - | - | - | - | - | - | - | - | - | - | - | - | 4.8 | 1.6 | - | - | - | - | - | - | 3.2 | - | - |
|  | MID-R3 | 4.4 | - | - | - | - | - | - | - | - | - | - | - | - | - | - | - | - | - | - | - | - | 4.4 | - | - |
|  | MID-R7 | 8.0 | - | - | - | - | - | - | - | - | - | - | - | - | - | - | - | - | - | - | - | - | 8.0 | - | - |
|  | MID-H10 | 2.4 | - | - | - | - | - | 0.4 | 0.4 | - | - | - | - | - | 0.4 | - | 0.4 | 0.4 | - | - | - | - | 0.4 | - | - |
|  | MID-H11 | 3.6 | - | - | - | - | - | - | - | - | - | - | - | - | - | 0.4 | - | 3.2 | - | - | - | - | - | - | - |
|  | MID-R20 | 4.0 | - | - | - | - | - | - | - | - | - | - | - | - | - | 0.8 | - | 0.4 | - | - | - | - | 2.8 | - | - |
|  | MID-H21 | 44.4 | - | - | - | - | - | - | 2.0 | 37.6 | - | - | 4.8 | - | - | - | - | - | - | - | - | - | - | - | - |
| Kure | KUR-02 | 28.0 | - | - | - | - | - | 0.8 | - | - | - | - | - | - | - | - | - | 22.8 | - | - | - | - | 4.4 | - | - |
|  | KUR-09 | - | - | - | - | - | - | - | - | - | - | - | - | - | - | - | - | - | - | - | - | - | - | - | - |
|  | KUR-12 | 15.2 | - | - | - | - | - | 0.4 | - | - | - | - | - | - | - | - | - | 10.4 | - | - | - | - | 4.4 | - | - |
|  | KUR-14 | 10.0 | - | - | - | - | - | 3.2 | - | - | - | - | - | - | - | - | - | 2.8 | - | - | - | - | 4.0 | - | - |
|  | KUR-17 | 10.8 | - | - | - | - | - | 1.6 | 2.4 | 3.2 | - | - | - | - | - | - | - | 2.0 | - | - | - | 0.4 | 1.2 | - | - |
|  | KUR-18 | 15.2 | - | - | - | - | - | - | - | - | - | - | - | - | - | - | - | - | - | - | 15.2 | - | - | - | - |
|  | KUR-R33 | 14.8 | - | - | - | - | - | 0.4 | - | - | - | - | - | - | - | - | - | 13.6 | - | - | - | - | 0.4 | 0.4 | - |
|  | KUR-R35 | 1.6 | - | - | - | - | - | - | - | - | - | - | - | - | - | - | - | 1.6 | - | - | - | - | - | - | - |
|  | KUR-R36 | 9.2 | - | - | - | - | - | - | 0.4 | - | - | - | - | - | - | - | - | 6.0 | 0.8 | - | - | - | 2.0 | - | - |

Table S3: Percent cover of scleractinian coral species by site. Metadata for each site is presented in Table S1. Sum totals for each row equal the percent cover of coral recorded in Table S4.
