# Supplementary material for: Benthic Composition of a Healthy Subtropical Reef: Baseline Species-Level Cover, with an Emphasis on Algae, in the Northwestern Hawaiian Islands
Source: PLoS One. 2010 Mar 17;5(3):e9733. doi: 10.1371/journal.pone.0009733 (PMC2840019; doi:10.1371/journal.pone.0009733)
Supplement: Table S4 — Percent cover of benthic functional groups at sites in the Northwestern Hawaiian Islands. Metadata for each site is presented in Table S1. Percent cover of macroalgal and scleractinian coral species are presented in Tables S2 and S3. (0.15 MB DOC) [file pone.0009733.s004.doc]

| Island | Site | Coral | Fleshy macroalgae | Calcified macroalgae | Turf algae | Crustose coralline red algae | Cyanobacteria | Zooanthid | Non-coral invertebrates | Sand |
| --- | --- | --- | --- | --- | --- | --- | --- | --- | --- | --- |
| FFS | FFS-H6 | 36.4 | 7.2 | 15.2 | 40.4 | - | - | - | 0.8 | - |
|  | FFS-12 | 81.6 | 0.4 | 0.4 | 11.2 | 6.4 | - | - | - | - |
|  | FFS-21 | 59.2 | 0.4 | 6 | 29.6 | 4.8 | - | - | - | - |
|  | FFS-22 | 1.6 | 36 | 0.8 | 29.2 | 7.2 | - | - | - | 25.2 |
|  | FFS-25 | 20.0 | 37.6 | 15.6 | 24.4 | 1.6 | - | - | - | 0.8 |
|  | FFS-29 | 21.6 | 25.2 | 3.2 | 43.2 | 4.0 | - | 0.4 | - | 2.4 |
|  | FFS-R29 | 22.4 | 2.8 | 11.6 | 40.0 | 10.8 | 0.4 | - | - | 12.0 |
|  | FFS-30 | 24.8 | 2 | 29.6 | 34.4 | 8.0 | 0.4 | - | 0.4 | 0.4 |
|  | FFS-R30 | 0.8 | 17.2 | 2.8 | 70.4 | 8.0 | - | - | - | 0.8 |
|  | FFS-32 | 19.2 | 32.4 | 12.4 | 25.6 | 8.0 | - | - | - | 2.4 |
|  | FFS-33 | 26.8 | 20.4 | 8.8 | 33.6 | 2.0 | - | - | - | 8.4 |
|  | FFS-34 | 3.6 | 24.8 | 0.8 | 67.2 | 2.0 | - | - | 1.2 | 0.4 |
|  | FFS-35 | 52.8 | 10 | 15.2 | 16.8 | 3.6 | 0.4 | - | - | 1.2 |
|  | FFS-R46 | 42.0 | 10.4 | - | 40.0 | 6.8 | 0.4 | 0.4 | - | - |
| Maro Reef | MAR-R1 | 61.6 | 3.6 | 11.2 | 21.2 | 0.8 | 0.4 | - | - | 1.2 |
|  | MAR-R3 | 72.8 | 1.6 | 7.2 | 7.2 | 8.8 | 0.8 | 1.6 | - | - |
|  | MAR-08 | 37.6 | 0.4 | 14 | 38.8 | 7.6 | - | - | - | 1.6 |
|  | MAR-R12 | 23.6 | 9.6 | 22.8 | 30.0 | 10.0 | - | - | - | 4.0 |
|  | MAR-22 | 32.4 | - | 8.4 | 45.2 | 1.2 | 12.4 | - | - | 0.4 |
|  | MAR-32 | 20.4 | 0.4 | 16.8 | 45.6 | 16.8 | - | - | - | - |
| Laysan | LAY-05 | 7.6 | 9.2 | 44.8 | 16.0 | 21.2 | - | - | 0.4 | 0.8 |
|  | LAY-R9 | 24.0 | 16 | 33.2 | 14.4 | 4.4 | 7.2 | - | - | 0.8 |
|  | LAY-R12 | 12.8 | 11.6 | 39.6 | 23.2 | 11.6 | 1.2 | - | - | - |
| Lisianski | LIS-R7 | 59.2 | 12 | 6 | 17.6 | 5.2 | - | - | - | - |
|  | LIS-10 | 51.6 | 1.6 | 1.2 | 30.0 | 15.6 | - | - | - | - |
|  | LIS-R10 | 50.8 | 6.4 | 11.2 | 14.0 | 17.6 | - | - | - | - |
|  | LIS-12 | 5.2 | 13.6 | 34.8 | 18.8 | 27.2 | 0.4 | - | - | - |
|  | LIS-R14 | 45.6 | 8 | 21.2 | 10.8 | 13.2 | - | - | - | 1.2 |
|  | LIS-18 | 18.0 | 30.8 | 23.6 | 18.0 | 8.4 | - | - | - | 1.2 |
| PHR | PHR-22 | 3.2 | 60.8 | 3.6 | 20.4 | 0.8 | - | - | 0.8 | 10.4 |
|  | PHR-23 | 2.0 | 16.4 | 47.6 | 15.2 | 18.0 | - | - | - | 0.8 |
|  | PHR-24 | 8.0 | 1.2 | 63.2 | - | 0.8 | 25.6 | - | 0.4 | 0.8 |
|  | PHR-26 | 34.8 | 18.4 | 3.2 | 37.2 | 6.4 | - | - | - | - |
|  | PHR-R26 | 6.8 | 62.8 | 9.6 | 10.0 | 0.8 | - | 0.4 | 2.0 | 7.6 |
|  | PHR-30 | 6.0 | 52.8 | 5.6 | 28.8 | 1.2 | - | - | 1.2 | 4.4 |
|  | PHR-31 | 36.4 | 10.8 | 3.6 | 20.0 | - | 0.4 | - | - | 28.8 |
|  | PHR-R31 | 62.0 | - | - | 36.4 | 0.4 | - | - | 1.2 | - |
|  | PHR-32 | 3.6 | 78.8 | 1.6 | 6.0 | - | - | - | - | 10.0 |
|  | PHR-R32 | 0.8 | 20.8 | 5.2 | 58.8 | 2.4 | - | - | 0.4 | 11.6 |
|  | PHR-33 | 1.6 | 80.4 | 2 | 12.8 | 1.6 | - | - | - | 1.6 |
|  | PHR-34 | 18.4 | 10 | 6 | 62.8 | 1.2 | - | - | 1.6 | - |
|  | PHR-R39 | 1.6 | 9.6 | 13.6 | 43.6 | 31.2 | 0.4 | - | - | - |
|  | PHR-R42 | 5.2 | 80 | 3.2 | 8.0 | 0.8 | - | - | 1.6 | 1.2 |
|  | PHR-R44 | 21.2 | 0.8 | 21.2 | 48.8 | 7.2 | - | 0.8 | - | - |
| Midway | MID-01 | 48.4 | 9.2 | - | 37.2 | 2.8 | 0.8 | - | - | 1.6 |
|  | MID-02 | 1.2 | 34 | 10.8 | 15.6 | - | - | - | 0.4 | 38.0 |
|  | MID-03 | 9.6 | 0.8 | - | 84.8 | 1.6 | - | - | 0.8 | 2.4 |
|  | MID-R3 | 4.4 | 12 | 0.4 | 75.2 | 2.8 | 3.6 | - | 1.2 | 0.4 |
|  | MID-R7 | 8.0 | 38.4 | 0.8 | 50.8 | 0.4 | - | - | 0.8 | 0.8 |
|  | MID-H10 | 2.4 | 36 | 0.8 | 52.8 | 1.6 | 2.0 | - | - | 4.4 |
|  | MID-H11 | 3.6 | 42.8 | 6.4 | 18.0 | 3.6 | 25.2 | - | 0.4 | - |
|  | MID-R20 | 4.0 | 47.2 | 1.6 | 34.4 | 6.4 | 0.4 | - | - | 6.0 |
|  | MID-H21 | 44.4 | 6.4 | 0.4 | 40.0 | 2.4 | 0.8 | - | - | 5.6 |
| Kure | KUR-02 | 28.0 | 44.8 | 2 | 18.0 | 6.0 | - | 0.8 | 0.4 | - |
|  | KUR-09 | - | 64 | 1.2 | 24.4 | 6.8 | - | - | - | 3.6 |
|  | KUR-12 | 15.2 | 63.2 | 1.2 | 17.2 | 3.2 | - | - | - | - |
|  | KUR-14 | 10.0 | 31.2 | - | 51.2 | 4.8 | - | - | - | 2.8 |
|  | KUR-17 | 10.8 | 21.2 | - | 53.6 | 8.0 | 0.4 | - | - | 6.0 |
|  | KUR-18 | 15.2 | 61.2 | - | 18.0 | 0.4 | - | - | - | 5.2 |
|  | KUR-R33 | 14.8 | 34.4 | 2 | 41.2 | 7.2 | - | - | - | 0.4 |
|  | KUR-R35 | 1.6 | 65.2 | 1.2 | 26.0 | 4.8 | 0.4 | - | - | 0.8 |
|  | KUR-R36 | 9.2 | 21.2 | 1.2 | 53.6 | 4.4 | - | - | - | 10.4 |

Table S4: Percent cover of benthic functional groups at sites in the Northwestern Hawaiian Islands. Metadata for each site is presented in Table S1. Percent cover of macroalgal and scleractinian coral species are presented in Tables S2 and S3.
