# Supplementary material for: Benthic Composition of a Healthy Subtropical Reef: Baseline Species-Level Cover, with an Emphasis on Algae, in the Northwestern Hawaiian Islands
Source: PLoS One. 2010 Mar 17;5(3):e9733. doi: 10.1371/journal.pone.0009733 (PMC2840019; doi:10.1371/journal.pone.0009733)
Supplement: Table S5 — Average percent cover of the six most dominant items occurring at forereefs on each island by Quadrant (NW, NE, SE, SW). Average percent cover is followed by standard error in parentheses. “n” = the number of sites surveyed in each island/quadrant, “ave. sim.” = average similarity (as determined through SIMPER analyses) of sites within each island/quadrant. (0.08 MB DOC) [file pone.0009733.s005.doc]

|  | **NW** |  | **NE** |  | **SE** |  | **SW** |  |
| --- | --- | --- | --- | --- | --- | --- | --- | --- |
|  | *n* = 1 |  | *n* = 1 |  | *n* = 2, ave. sim. = 54.80 |  |  |  |
| **FFS** | turf algae  *Porites lobata*  *Halimeda velasquezii*  *Acropora cytherea*  *Lobophora variegata*  *Microdictyon setchellianum* | 40.4  22.0  12.0  10.4  3.2  3.2 | turf algae  *Lobophora variegata*  *Pocillopora meandrina*  crustose coralline red algae  *Halimeda velasquezii*  Sand | 43.2  23.6  15.6  4.0  3.2  2.4 | turf algae  *Microdictyon setchellianum*  *Lobophora variegata*  *Porites lobata*  *Halimeda velasquezii*  *Pocillopora meandrina* | 45.8 (21.4)  23.2 (6.8)  8.0 (0.4)  7.8 (6.2)  6.2 (5.8)  3.8 (1.8) | No southwest forereef sites visited |  |
|  | *n* = 4, ave. sim. = 50.00 |  |  |  | *n* = 2, ave. sim. = 66.80 |  |  |  |
| **Maro** | turf algae  *Porites lobata*  *Montipora capitata*  *Porites compressa*  *Halimeda velasquezii*  crustose coralline red algae | 24.3 (6.7)  15.6 (1.4)  12.4 (4.3)  9.9 (4.8)  8.0 (3.0)  5.4 (1.8) | No northeast forereef sites visited |  | turf algae  *Porites compressa*  crustose coralline red algae  *Halimeda opuntia*  Cyanobacteria  *Montipora capitata* | 45.4 (0.2)  14.8 (0.4)  9.0 (7.8)  7.0 (6.2)  6.2 (6.2)  5.4 (5.4) | No southwest forereef sites visited |  |
|  | *n* = 1 |  | *n* = 1 |  |  |  | n = 1 |  |
| **Laysan** | *Halimeda velasquezii*  turf algae  crustose coralline red algae  *Porites lobata*  *Microdictyon setchellianum*  *Laurencia majuscula* | 37.6  23.2  10.0  10.0  6.4  2.8 | *Halimeda velasquezii*  crustose coralline red algae  turf algae  *Laurencia majuscula*  *Pocillopora meandrina*  Sand | 44.0  21.2  16.0  8.4  7.2  0.8 | No southeast forereef sites visited |  | *Halimeda velasquezii*  *Porites lobata*  turf algae  *Laurencia majuscula*  Cyanobacteria  crustose coralline red algae | 32.8  20.4  14.4  11.2 7.2  4.4 |
|  | *n* = 3, ave. sim. = 61.20 |  |  |  | *n* = 1 |  | n = 2, ave. sim. = 66.40 |  |
| **Lisianski** | *Porites lobata*  *Halimeda opuntia*  turf algae  *Microdictyon setchellianum*  *Halimeda velasquezii*  crustose coralline red algae | 16.8 (7.2)  16.3 (3.9)  15.9 (2.5)  11.7 (5.1)  10.1 (0.1)  9.2 (1.6) | No northeast forereef sites visited |  | *Porites evermanni*  turf algae  crustose coralline red algae  *Porites compressa*  *Montipora capitata*  *Lobophora variegata* | 33.2  30.0  15.6  9.6  4.0  1.2 | *Porites evermanni*  turf algae  crustose coralline red algae  *Porites compressa*  *Porites lobata*  *Montipora patula* | 26.4 (6.4)  15.8 (1.8)  11.4 (6.2)  9.8 (1.0)  7.4 (2.6)  5.8 (3.4) |
|  | *n* = 2, ave. sim. = 64.80 |  |  |  | *n* = 2, ave. sim. = 75.20 |  | n = 2, ave. sim. = 20.00 |  |
| **PHR** | turf algae  crustose coralline red algae  *Halimeda velasquezii*  *Porites lobata*  *Lobophora variegata*  *Halimeda discoidea*  *Palythoa caesia*  *Pocillopora ligulata* | 46.2 (2.6)  19.2 (12.0)  16.8 (3.6)  10.4 (10.4)  4.8 (4.0)  0.4 (0.4)  0.4 (0.4)  0.4 (0.4) | No northeast forereef sites visited |  | *Microdictyon setchellianum*  turf algae  Sand  *Halimeda velasquezii*  *Porites lobata*  *Lobophora variegata* | 67.2 (8.8)  11.4 (1.4)  4.6 (3.0)  4.0 (3.6)  3.6 (2.4)  2.6 (1.0) | *Microdictyon setchellianum*  turf algae  *Lobophora variegata*  *Pocillopora ligulata*  *Porites lobata*  *Halimeda velasquezii* | 37.4 (37.4)  35.4 (27.4)  7.6 (2.4)  5.8 (4.6)  3.4 (1.4)  3.4 (2.6) |
|  |  |  |  |  | *n* = 1 |  | n = 3, ave. sim. = 62.00 |  |
| **Midway** | No northwest forereef sites visited |  | No northeast forereef sites visited |  | turf algae  Sand  *Microdictyon setchellianum*  *Padina* sp.  *Dictyota ceylanica*  *Pocillopora meandrina* | 15.6  38.0  25.2  10.8  7.6  0.8 | turf algae  *Dictyota ceylanica*  *Laurencia galtsoffii*  *Lobophora variegata*  *Porites lobata*  Sand  Cyanobacteria | 59.6 (7.8)  12.0 (11.2)  9.2 (9.2)  7.6 (2.0)  4.3 (2.2)  1.9 (1.3)  1.9 (1.0) |
|  | *n* = 1 |  |  |  | *n* = 1 |  | n = 1 |  |
| **Kure** | *Microdictyon setchellianum*  *Pocillopora meandrina*  turf algae  *Laurencia galtsoffii*  crustose coralline red algae  *Lobophora variegata* | 28.8  22.8  18.0  6.0  6.0  6.0 | No northeast forereef sites visited |  | *Microdictyon setchellianum*  turf algae  *Stypopodium flabelliforme*  *Pocillopora meandrina*  *Lobophora variegata*  *Laurencia galtsoffii* | 27.6  17.2  15.2  10.4  10.0  5.2 | turf algae  *Microdictyon setchellianum*  *Pocillopora meandrina*  *Lobophora variegata*  crustose coralline red algae  *Dictyota ceylanica* | 41.2  22.4  13.6  8.4  7.2  2.0 |

Table S5: Average percent cover of the six most dominant items occurring at forereefs on each island by Quadrant (NW, NE, SE, SW). Average percent cover is followed by standard error in parentheses. “*n*” = the number of sites surveyed in each island/quadrant, ”ave. sim.” = average similarity (as determined through SIMPER analyses) of sites within each island/quadrant.
